# Risk of arterial stiffness according to metabolically healthy obese phenotype: a combined cross-sectional and longitudinal study in kailuan cohort

**DOI:** 10.18632/aging.203075

**Published:** 2021-06-03

**Authors:** Anxin Wang, Yu Wang, Yingting Zuo, Xue Tian, Shuohua Chen, Yihan Ma, Xu Han, Shouling Wu, Xingquan Zhao

**Affiliations:** 1 China National Clinical Research Center for Neurological Diseases, Beijing Tiantan Hospital, Capital Medical University, Beijing, China; 2 Department of Neurology, Beijing Tiantan Hospital, Capital Medical University, Beijing, China; 3 Department of Epidemiology and Health Statistics, School of Public Health, Capital Medical University, Beijing, China; 4 Beijing Municipal Key Laboratory of Clinical Epidemiology, Beijing, China; 5 Department of Cardiology, Kailuan Hospital, North China University of Science and Technology, Tangshan, China; 6 Graduate School of North China University of Science and Technology, Tangshan, China; 7 Research Unit of Artificial Intelligence in Cerebrovascular Disease, Chinese Academy of Medical Sciences, Beijing, China

**Keywords:** obesity, metabolic syndrome, arterial stiffness, baPWV

## Abstract

We aim to investigate the risk of incident arterial stiffness according to metabolically healthy obese (MHO) phenotype in Chinese population. 37,180 participants with at least one-time measurement of branchial-ankle pulse wave velocity (baPWV) were included in the cross-sectional analysis, and 16,236 participants with repeated measurement of baPWV during the follow-ups were included in the longitudinal study. Cross-classification of body mass index (BMI) categories and metabolic health status created six groups. Linear and logistic regression analyses were used. The results of cross-sectional and longitudinal investigation were essentially the same, as the abnormality of baPWV increased with BMI categories in metabolically healthy participants, while the increasing tendency disappeared in metabolically unhealthy participants. A 1.4-fold, 2.2-fold increased risk for the new occurrence of arterial stiffness were documented in MHO and metabolically unhealthy obese participants compared to metabolically healthy normal-weight controls in the fully adjusted model. Further stratified analysis showed that metabolic health status was an interaction factor between BMI and arterial stiffness in all study populations (*P*=0.0001 for cross-sectional study and *P*=0.0238 for longitudinal study). In conclusion, metabolic health status and BMI categories contribute to the progression of arterial stiffness, while BMI is positively associated with arterial stiffness only in metabolically healthy participants.

## INTRODUCTION

Branchial-ankle pulse wave velocity (baPWV), a promising indicator of both central and peripheral arterial stiffness [1], has been proven to be strongly associated with cardio-cerebrovascular morbidity and mortality in a recent meta-analysis of 8 studies [2]. The subclinical state of atherosclerosis could be improved with baPWV-guided lifestyle modification and therapeutic intervention [3].

Metabolic syndrome (MetS) is recognized as a cluster of risk factors for atherosclerotic cardiovascular disease (CVD), including hypertension, hyperlipidemia, hyperglycemia, and broadened waist circumference (WC) [4, 5]. It is known that baPWV increases with MetS, as well as the number of MetS components [6–8]. Obesity, which has reached an epidemic level owing to economic development in China, often coexists with MetS [9]. While no consensus has been reached on the correlation between obesity and baPWV [10, 11]. Currently, a subset of obese individuals without MetS, identified as metabolically healthy obese (MHO), have attracted extensive attention due to the controversial results regarding cardiovascular risk. Some studies demonstrated that obesity status exerted no extra influence on CVD [12, 13]. Others indicated MHO was a transient condition from metabolically healthy to unhealthy phenotypes, and obesity carried an increased risk of CVD regardless of metabolic health status [14–16]. The development of clinical CVD events usually requires a long period of time, subclinical atherosclerosis as arterial stiffness might better estimate the impact of obesity or MetS on CVD within a short period of time. Considering the above-mentioned research, the role of obesity on the association between MetS and baPWV is worth further exploration.

Therefore, we aimed to investigate the risk of incident arterial stiffness according to MHO phenotype in Chinese population using the Kailuan cohort study.

## RESULTS

### Patients characteristics

In the cross-sectional analysis, out of the 37,180 enrolled participants, 10,295 (27.69%) were metabolically healthy normal weight (MH-NW), 7,171 (19.29%) were metabolically healthy overweight (MH-OW), 2,337 (6.29%) were MHO, 4,253 (11.44%) were metabolically unhealthy normal weight (MUH-NW), 8,314 (22.36%) were metabolically unhealthy overweight (MUH-OW), and 4,810 (12.94%) were metabolically unhealthy obese (MUO). The baseline characteristics of participants among the body mass index (BMI)-MetS categories were presented in Table 1. In addition to risk factors referred to MetS, the individuals in the metabolically unhealthy group were more likely to be older, male, less educated, a current smoker or alcoholic, and having slightly higher average income and salt intake. People who were overweight or obese had a larger WC than those with normal weight and this tendency was more pronounced in the metabolically unhealthy group.

**Table 1:** Characteristics of participants according to body mass index - metabolic health status at baseline.

**Variables**	**Metabolically healthy (n=19803)**				**Metabolically unhealthy (n=17377)**			***P*-value**
	**Normal weight**	**Overweight**	**Obese**		**Normal weight**	**Overweight**	**Obese**	
n (%)	10295 (27.69)	7171 (19.29)	2337 (6.29)		4253 (11.44)	8314 (22.36)	4810 (12.94)	
Age, years	52.27 ± 11.98	54.86 ± 11.54	53.94 ± 12.01		58.86 ± 11.74	59.00 ± 10.78	57.73 ± 11.11	<0.001
Male, n (%)	5715 (55.51)	5230 (72.93)	1777 (76.04)		3097 (72.82)	6634 (79.79)	3874 (80.54)	<0.001
High school or above, n (%)	4646 (45.17)	2750 (38.42)	854 (36.62)		1311 (30.88)	2570 (30.99)	1525 (31.75)	<0.001
Average income ≥ ¥1000/month, n (%)	5706 (59.82)	3916 (58.62)	1204 (54.85)		2461 (61.42)	4800 (61.66)	2818 (62.25)	<0.001
Current smoker, n (%)	2928 (28.46)	2393 (33.41)	778 (33.36)		1573 (37.03)	3137 (37.78)	1759 (36.59)	<0.001
Current alcoholic, n (%)	3178 (30.90)	2701 (37.71)	906 (38.85)		1688 (39.74)	3558 (42.85)	1950 (40.57)	<0.001
Physical activity ≥ 3 times/week, n (%)	1290 (12.54)	1058 (14.78)	317 (13.59)		634 (14.94)	1349 (16.26)	683 (14.22)	<0.001
Salt intake ≥12 g/day, n (%)	909 (8.84)	723 (10.10)	229 (9.82)		485 (11.43)	963 (11.61)	661 (13.76)	<0.001
BMI, kg/m^2^	21.71 ± 1.45	25.72 ± 1.12	30.11 ± 2.17		22.30 ± 1.27	25.97 ± 1.13	30.30 ± 2.18	<0.001
WC, cm	79.49 ± 8.20	87.45 ± 7.74	93.39 ± 9.90		83.88 ± 8.40	90.13 ± 7.85	97.29 ± 9.44	<0.001
Systolic blood pressure, mmHg	117.33 ± 15.70	122.82 ± 16.16	94.27 ± 9.40		134.99 ± 17.11	137.01 ± 16.74	139.26 ± 16.58	<0.001
Diastolic blood pressure, mmHg	77.29 ± 9.47	80.92 ± 9.61	83.46 ± 10.45		86.28 ± 10.02	88.05 ± 9.89	90.36 ± 10.08	<0.001
Fasting glucose, mmol/L	5.05 ± 0.81	5.16 ± 0.91	5.15 ± 0.88		6.18 ± 1.79	6.35 ± 1.93	6.44 ± 1.97	<0.001
Total cholesterol, mmol/L	4.78 ± 1.35	4.87 ± 1.25	4.86 ± 0.86		5.20 ± 1.72	5.21 ± 1.17	5.19 ± 1.03	<0.001
Triglycerides, mmol/L	1.08 ± 0.62	1.29 ± 0.76	1.35 ± 0.75		2.04 ± 1.44	2.30 ± 1.50	2.45 ± 1.48	<0.001
LDL-cholesterol, mmol/L	2.42 ± 0.77	2.57 ± 0.85	2.60 ± 0.71		2.69 ± 1.44	2.72 ± 0.91	2.72 ± 1.13	<0.001
HDL-cholesterol, mmol/L	1.66 ± 0.46	1.57 ± 0.41	1.51 ± 0.38		1.53 ± 0.49	1.46 ± 0.45	1.41 ± 0.42	<0.001
CRP, mg/L	1.53 ± 3.65	2.02 ± 4.86	2.57 ± 5.50		2.19 ± 4.85	2.42 ± 4.18	3.05 ± 6.01	<0.001
Hypertension, n (%)	490 (4.76)	619 (8.63)	279 (11.94)		898 (21.11)	2319 (27.89)	1574 (32.72)	<0.001
Hypertension medication, n (%)	360 (3.50)	443 (6.18)	208 (8.90)		674 (15.85)	1763 (21.21)	1214 (25.24)	<0.001
Diabetes, n (%)	89 (0.86)	109 (1.52)	27 (1.16)		367 (8.63)	973 (11.70)	532 (11.06)	<0.001
Diabetes medication, n (%)	69 (0.67)	82 (1.14)	20 (0.86)		303 (7.12)	760 (9.14)	418 (8.69)	<0.001
Dyslipidemia, n (%)	134 (1.30)	138 (1.92)	39 (1.67)		407 (9.57)	1023 (12.30)	7.04 (14.64)	<0.001
Dyslipidemia medication, n (%)	36 (0.35)	24 (0.33)	14 (0.60)		129 (3.03)	321 (3.86)	258 (5.36)	<0.001
History of MI	36 (0.35)	54 (0.51)	12 (0.51)		50 (1.18)	115 (1.39)	91 (1.89)	<0.001
History of stroke	62 (0.60)	56 (0.73)	17 (0.73)		80 (1.88)	189 (2.28)	84 (1.75)	<0.001

Values are (%) for categorical variables and mean ± SD or median (IQR) for continuous variables; BMI, body mass index; WC, waist circumstance; LDL, low-density lipoprotein; HDL, high-density lipoprotein; CRP, C-reactive protein.

### Cross-sectional investigation

8,182 cases of arterial stiffness were documented based on the first measurement of baPWV. Table 2 shows the odds ratios (ORs) for arterial stiffness stratified by BMI-MetS phenotypes. In the univariate analysis, the OR values were higher in other subgroups when compared with the MH-NW group, 1.35 (95% confidence interval [CI] 1.23-1.47) for MH-OW group, 1.46 (95%CI 1.29-1.65) for MHO group, 3.75 (95%CI 3.43-4.09) for MUH-NW group, 3.72 (95%CI 3.45-4.01) for MUH-OW group, and 3.08 (95%CI 2.83-3.36) for MUO group. In the multivariate analysis, similar results were obtained after adjusting for potential covariates in all three models. When taking baPWV as a continuous variable rather than dichotomous variable, significant β coefficients were obtained as well (*P* < 0.001, Table 3). It was notable that, in the context of metabolically healthy participants, the abnormality of baPWV increased with BMI categories. Whereas, the increasing tendency was not pronounced in metabolically unhealthy participants.

**Table 2:** Odds ratios and 95%CI for risk of baPWV ≥ 1800 cm/s according to the body mass index – metabolic health status.

	**MH-NW**	**MH-OW**	**MHO**	**MUH-NW**	**MUH-OW**	**MUO**
baPWV ≥ 1800 cm/s, n (%)	1198 (11.64)	1080 (15.06)	377 (16.13)	1405 (33.04)	2733 (32.87)	1389 (28.88)
Univariate analysis	Ref.	1.35 (1.23,1.47)	1.46 (1.29,1.65)	3.75 (3.43,4.09)	3.72 (3.45,4.01)	3.08 (2.83,3.36)
Multivariate analysis						
Model 1	Ref.	1.10 (1.10,1.10)	1.28 (1.10,1.46)	2.63 (2.38,2.90)	2.55 (2.35,2.78)	2.26 (2.05,2.48)
Model 2	Ref.	1.10 (0.99,1.21)	1.26 (1.09,1.44)	2.58 (2.34,2.84)	2.52 (2.31,2.74)	2.24 (2.04,2.47)
Model 3	Ref.	1.09 (0.99,1.20)	1.24 (1.08,1.43)	2.56 (2.32,2.82)	2.50 (2.29,2.72)	2.20 (2.00,2.43)
Sensitivity analysis*	Ref.	1.11 (1.00-1.22)	1.26 (1.09-1.44)	2.62 (2.37-2.90)	2.53 (2.32-2.76)	2.22 (2.01-2.45)

Data are OR (95% CI) unless otherwise stated.

baPWV, branchial-ankle pulse wave velocity; MH-NW, metabolically healthy normal weight; MH-OW, metabolically healthy overweight; MHO, metabolically healthy obese; MUH-NW, metabolically unhealthy normal weight; MUH-OW, metabolically unhealthy overweight; MUO, metabolically unhealthy obese.

Model 1 adjusted for age and sex.

Model 2 adjusted for variates in model 1 plus educational level, average income, smoking, drinking, physical activity, sodium intake, history of myocardial infarction, and history of stroke.

Model 3 adjusted for variates in model 2 plus C-reactive protein.

*Sensitivity analysis excluded 811 participants with a history of cardiovascular disease.

**Table 3:** Risk of baPWV according to the body mass index – metabolic health status.

	**MH-NW**	**MH-OW**	**MHO**	**MUH-NW**	**MUH-OW**	**MUO**
baPWV, m/s	14.28 ± 3.25	15.01 ± 3.22	15.15 ± 3.22	16.97 ± 4.05	17.03 ± 3.81	16.61 ± 3.46
Univariate analysis	Ref.	0.74 (0.64,0.85)	0.88 (0.72,1.03)	2.69 (2.57,2.83)	2.76 (2.66,2.86)	2.33 (2.21,2.45)
Multivariate analysis						
Model 1	Ref.	0.16 (0.07,0.25)	0.39 (0.26,0.53)	1.53 (1.43,1.65)	1.49 (1.41,1.59)	1.24 (1.14,1.35)
Model 2	Ref.	0.16 (0.06,0.25)	0.37 (0.24,0.51)	1.52 (1.41,1.62)	1.48 (1.41,1.57)	1.23 (1.12,1.33)
Model 3	Ref.	0.14 (0.05,0.24)	0.35 (0.21,0.49)	1.50 (1.39,1.61)	1.46 (1.37,1.55)	1.19 (1.09,1.30)
Sensitivity analysis*	Ref.	0.16 (0.06,0.25)	0.37 (0.24,0.51)	1.53 (1.42,1.64)	1.47 (1.38,1.56)	1.22 (1.11,1.33)

Data are β - coefficients (95% CI) unless otherwise stated.

baPWV, branchial-ankle pulse wave velocity; MH-NW, metabolically healthy normal weight; MH-OW, metabolically healthy overweight; MHO, metabolically healthy obese; MUH-NW, metabolically unhealthy normal weight; MUH-OW, metabolically unhealthy overweight; MUO, metabolically unhealthy obese.

Model 1 adjusted for age and sex.

Model 2 adjusted for variates in model 1 plus educational level, average income, smoking, drinking, physical activity, sodium intake, history of myocardial infarction, and history of stroke.

Model 3 adjusted for variates in model 2 plus C-reactive protein.

*Sensitivity analysis excluded 811 participants with a history of cardiovascular disease.

### Longitudinal investigation

16,236 participants with follow-up measurements of baPWV were eventually included in the longitudinal study. The average increases of baPWV were 0.35 m/s, 0.42 m/s, 0.36 m/s, 0.43m/s, 0.31m/s, and 0.40 m/s in the six BMI-MetS phenotypes over the follow-up period. In comparison with MH-NW group, crude β coefficients per risk category increase were 0.27 (95%CI 0.16-0.38), 0.24 (95%CI 0.07-0.41), 0.66 (95%CI 0.52-0.80), 0.64 (95%CI 0.52-0.75), and 0.62 (95%CI 0.48-0.75), respectively. The results of multivariate analysis, in which conventional risk factors were adjusted, shown robust consistency with univariate analysis (*p* < 0.001, Table 4).

**Table 4:** Risk of change of baPWV according to the body mass index – metabolic health status.

	**MH-NW** **(n=5036)**	**MH-OW** **(n=3244)**	**MHO** **(n=993)**	**MUH-NW** **(n=1671)**	**MUH-OW** **(n=3398)**	**MUO** **(n=1894)**
Change of baPWV, m/s	0.35 (-0.53,1.35)	0.42 (-0.55,1.52)	0.36 (-0.76,1.53)	0.43 (-0.82,1.87)	0.31 (-0.92,1.70)	0.40 (-0.85,1.79)
Univariate analysis	Ref.	0.27 (0.16,0.38)	0.24 (0.07,0.41)	0.66 (0.52,0.80)	0.64 (0.52,0.75)	0.62 (0.48,0.75)
Multivariate analysis						
Model 1	Ref.	0.14 (0.04,0.25)	0.19 (0.03,0.36)	0.52 (0.38,0.66)	0.46 (0.34,0.57)	0.49 (0.36,0.62)
Model 2	Ref.	0.14 (0.04,0.25)	0.19 (0.02,0.35)	0.52 (0.38,0.65)	0.45 (0.35,0.57)	0.49 (0.36,0.62)
Model 3	Ref.	0.14 (0.04,0.25)	0.18 (0.02,0.35)	0.51 (0.38,0.65)	0.46 (0.35,0.57)	0.49 (0.36,0.62)
Model 3 with IPW*	Ref.	0.17 (0.10,0.24)	0.23 (0.11,0.34)	0.58 (0.43,0.72)	0.56 (0.44,0.68)	0.64 (0.50,0.77)
Sensitivity analysis**†**	Ref.	0.14 (0.02,0.24)	0.18 (0.01,0.35)	0.49 (0.35,0.63)	0.44 (0.33,0.56)	0.45 (0.32,0.59)

Data are β - coefficients (95% CI) unless otherwise stated.

baPWV, branchial-ankle pulse wave velocity; MH-NW, metabolically healthy normal weight; MH-OW, metabolically healthy overweight; MHO, metabolically healthy obese; MUH-NW, metabolically unhealthy normal weight; MUH-OW, metabolically unhealthy overweight; MUO, metabolically unhealthy obese; IPW, inverse probability weighting.

Model 1 adjusted for age and sex.

Model 2 adjusted for variates in model 1 plus educational level, average income, smoking, drinking, physical activity, sodium intake, history of myocardial infarction, and history of stroke.

Model 3 adjusted for variates in model 2 plus C-reactive protein.

*Shown is the primary analysis with an odds ratio from the multivariable logistic regression model with the same strata and covariates with IPTW according to the propensity score. The analysis included all the patients with baseline baPWV.

^†^Sensitivity analysis excluded 275 participants with a history of cardiovascular disease.

44.80% (7,273/16,236) participants of the cohort study had a normal baPWV at baseline, amongst them 1,419 cases of new occurrence arterial stiffness were identified during the 10-year follow-up period. The rate of new abnormal baPWV events increased gradually from MH-NW to MUO phenotype. After adjustment for age and sex, the growth trend remained in metabolically healthy participants, but not in metabolically unhealthy participants. Model 2 and model 3 showed consistent results with model 1. In the fully adjusted model, a 1.4-fold, 2.2-fold increased risk for the new occurrence of arterial stiffness were documented in MHO and MUO compared to MH-NW controls. The results of sensitivity analysis excluding participants with a history of CVD remained statistically significant. Additional information was given in Table 5.

**Table 5:** Odds ratios and 95%CI for risk of new occurrence of baPWV abnormality (baPWV≥ 1800 cm/s) according to the body mass index – metabolic health status.

	**MH-NW**	**MH-OW**	**MHO**	**MUH-NW**	**MUH-OW**	**MUO**
baPWV ≥ 1800 cm/s, n (%)	257 (5.55)	245 (8.46)	76 (8.61)	198 (16.43)	414 (16.63)	229 (15.58)
Univariate analysis	Ref.	1.57 (1.31,1.88)	1.60 (1.23,2.09)	3.34 (2.74,4.07)	3.39 (2.88,4.00)	3.14 (2.60,3.79)
Multivariate analysis						
Model 1	Ref.	1.24 (1.03,1.50)	1.37 (1.04,1.80)	2.34 (1.90,2.88)	2.24 (1.88,2.66)	2.22 (1.82,2.71)
Model 2	Ref.	1.25 (1.04,1.51)	1.27 (1.04,1.80)	2.31 (1.87,2.84)	2.25 (1.89,2.67)	2.21 (1.81,2.70)
Model 3	Ref.	1.25 (1.04,1.52)	1.37 (1.03,1.80)	2.31 (1.87,2.84)	2.25 (1.89,2.67)	2.21 (1.81,2.70)
Model 3 with IPW*	Ref.	1.25 (1.14,1.37)	1.46 (1.27,1.66)	2.22 (1.94,2.54)	2.25 (2.03,2.50)	2.35 (2.09,2.64)
Sensitivity analysis**†**	Ref.	1.22 (1.01,1.46)	1.41 (1.08,1.84)	2.29 (1.87,2.81)	2.21 (1.87,2.62)	2.12 (1.75,2.58)

Data are OR (95% CI) unless otherwise stated.

baPWV, branchial-ankle pulse wave velocity; MH-NW, metabolically healthy normal weight; MH-OW, metabolically healthy overweight; MHO, metabolically healthy obese; MUH-NW, metabolically unhealthy normal weight; MUH-OW, metabolically unhealthy overweight; MUO, metabolically unhealthy obese; IPW, inverse probability weighting.

Model 1 adjusted for age and sex.

Model 2 adjusted for variates in model 1 plus educational level, average income, smoking, drinking, physical activity, sodium intake, history of myocardial infarction, and history of stroke.

Model 3 adjusted for variates in model 2 plus C-reactive protein.

*Shown is the primary analysis with an odds ratio from the multivariable logistic regression model with the same strata and covariates with IPTW according to the propensity score. The analysis included all the patients with baseline baPWV.

^†^Sensitivity analysis excluded 275 participants with a history of cardiovascular disease.

### Stratified analysis

Metabolic health status was an interaction factor between BMI and arterial stiffness in all study participants (*P*=0.0001 for cross-sectional study and *P*=0.0238 for longitudinal study). In metabolically health participants, BMI demonstrated a dose-dependent increase in the risk of abnormal baPWV, with adjusted ORs of 1.07 (95%CI 0.97-1.18), 1.22 (95%CI 1.06-1.41) in the overweight and obese group. By contrast, no relation was found in participants with MetS in the cross-sectional study. These results were further validated in the cohort study (Table 6).

**Table 6:** Odds ratio and 95%CI for risk of baPWV≥ 1800 cm/s according to the body mass index stratified by different metabolic health status.

	**Normal weight**	**Overweight**	**Obese**	***P* for interaction**
Baseline of baPWV ≥ 1800 cm/s, n (%)				
MH	Ref.	1.07 (0.97,1.18)	1.22 (1.06,1.41)	0.0001
MUH	Ref.	0.98 (0.90,1.07)	0.86 (0.78,0.95)	
New occurrence of baPWV ≥ 1800 cm/s, n (%)				
MH	Ref.	1.19 (0.98,1.44)	1.30 (1.02,1.72)	0.0238
MUH	Ref.	0.99 (0.82,1.21)	0.98 (0.79,1.22)	

Data are OR (95% CI) unless otherwise stated.

baPWV, branchial-ankle pulse wave velocity; MH, metabolically healthy; MUH, metabolically unhealthy.

Adjusted for age, sex, educational level, average income, smoking, drinking, physical activity, sodium intake, history of myocardial infarction, history of stroke, and C-reactive protein.

## DISCUSSION

This study assessed the cross-sectional and longitudinal associations between BMI-MetS phenotypes and baPWV (either as a continuous or dichotomous variable). We found that metabolic health status and BMI categories contributed to the progression of arterial stiffness synchronously, and MHO was an intermediate stage between metabolically healthy and unhealthy status, instead of a benign phenotype. Moreover, BMI categories were correlated with abnormal baPWV in metabolically healthy participants. While the relationship disappeared in metabolically unhealthy participants, for whom MetS itself was the dominant risk factor for arterial stiffness.

The prevalence of MHO in the Chinese population varied from 4.2% to 11.4% due to the heterogeneous definition [16, 17]. In our study, the prevalence of MHO was 6.29% in the general population and 32.7% in obese subjects. Previous studies focused on the relationship between MHO and the risk of CVD. According to the research results, MetS and its components (hypertension, glucose intolerance, and dyslipidemia) are all documented independent risk factors for baPWV [7]. However, the impact of obesity on baPWV showed inconsistent results. A cross-sectional study observed a positive correlation between central obesity and arterial stiffness in China [6]. Lin L et al. demonstrated that transient MHO conferred an increased risk of abnormal baPWV [18]. Several studies reported that obese individuals had youthful arteries with lower PWV [19, 20]. By contrast, some studies found an irrelevant association between them [21, 22]. Obesity often coexists with other risk factors and accelerates arterial stiffness through its associated metabolic abnormalities. Although MetS is served as a risk enhancer, it is difficult to predict CVD risk quantitatively due to the mediating role of obesity. To take the contribution of obesity and other cardiometabolic risk factors separately, we thus used a modified harmonized IDF-MetS definition and subdivided it by the degree of obesity. The present study indicated that obesity did interact with metabolic status and BMI was positively associated with baPWV only in metabolically healthy participants.

It is noteworthy that BMI cannot fully reflect body composition and adiposity distribution. Those with excess visceral fat exhibited a greater risk of CVD than those with subcutaneous fat. WC is a more reliable index capable of differentiating between overall adiposity and abdominal adiposity among the same BMI range [23]. In our study, a significant positive correlation between WC and BMI categories was observed, which could partially offset the inadequacies of BMI. Beyond that, the issue of obesity paradox has aroused great concern. Although obesity contributes to the development of CVD, the long-term prognosis of obese individuals is often better due to their superior cardiorespiratory fitness against acute stress [24]. In terms of pathophysiological mechanisms, there was no paradoxical association between obesity and subclinical CVD as adipose tissue could impair vascular function through specific hormones and proinflammatory cytokines [25].

The strength of this study is its combined cross-sectional and longitudinal aspects. Nonetheless, there are still some limitations. First, the harmless feature of metabolically healthy phenotype was hung in doubt given the definition of MetS. There were 75.9% of MHO participants having one metabolic risk factor in our study. which could exert an additional effect on baPWV apart from obesity. Secondly, accumulated evidence indicated that MHO was not only an intermediate-stage, but also a transit condition from metabolically healthy to unhealthy status [14, 15]. Because of the relatively short follow-up time, the conversion of MHO status was not included in our statistics. Further study is needed to provide insight into the dynamic relationship between metabolically healthy obese phenotype and arterial stiffness.

In conclusion, both metabolic health status and BMI categories contribute to the progression of arterial stiffness, while BMI is positively associated with arterial stiffness only in metabolically healthy participants due to its fully mediating role through associated metabolic risk factors. Moreover, MHO is an intermediate stage between metabolically healthy and unhealthy status rather than a benign status, which highlights the need for active weight reduction and risk factor management.

## MATERIALS AND METHODS

### Ethics

The study was conducted in accordance with guidelines from the Helsinki Declaration and was approved by the Ethics Committees of both Kailuan General Hospital and Beijing Tiantan Hospital. All participants or their legal representatives provided written informed consent (Trial registration: ChiCTR-TNRC-11001489).

### Study population

**Figure 1:**
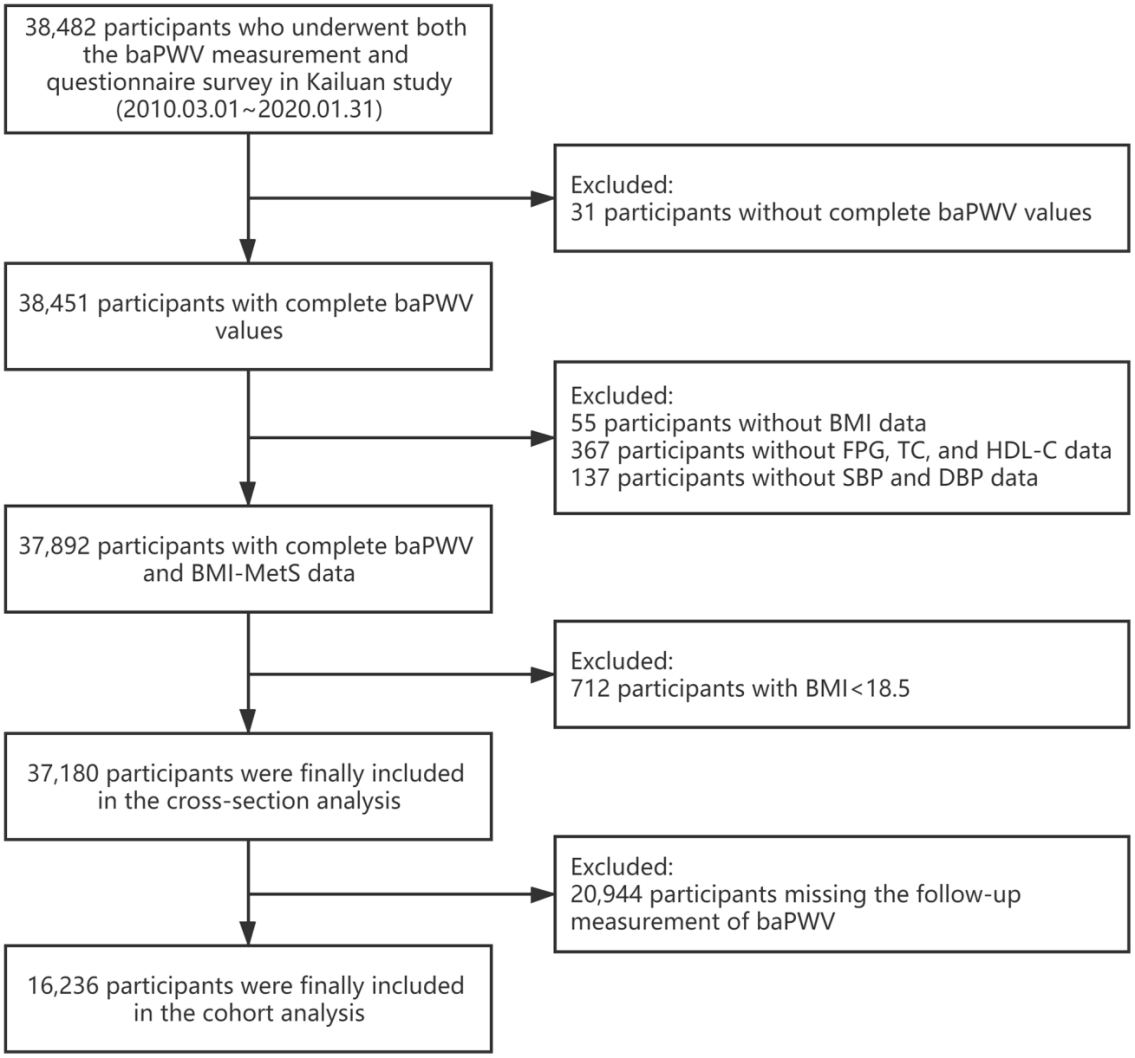
**Flow chart for selection of study participants.** baPWV, branchial-ankle pulse wave velocity; BMI, body mass index; FBG, fasting blood glucose; TC, total cholesterol; HDL-C, high-density lipoprotein cholesterol; SBP, systolic blood pressure; DBP, diastolic blood pressure; MetS, metabolic syndrome.

The Kailuan study is an ongoing prospective cohort study, details about the design and methods of this study have been published in detail previously [26]. From March 1, 2010, to January 31, 2020, 38,482 participants underwent both the baPWV measurement and questionnaire survey in the Kailuan cohort study. Among them, 31 participants without complete baPWV values and 559 participants without complete data regarded to BMI or MetS were excluded. Additionally, 712 individuals with a BMI below 18.5 kg/m^2^ were excluded. A total of 37,180 participants with at least one-time measurement of baPWV were included in the cross-sectional analysis. 16,236 participants, with repeated measurement of baPWV during a median follow-ups period of 2.8 years, were further included in the longitudinal study (Figure 1). Baseline characteristics between follow-up and lost-to-follow-up participants were shown in Supplementary Table 1.

### Definitions of obesity, metabolic syndrome, and metabolically healthy obese phenotype

BMI was calculated as weight divided by the square of height (kg/m^2^). According to the Working Group on Obesity in China, BMI was categorized as normal weight (18.5≤BMI<24.0 kg/m^2^), overweight (24.0≤BMI<28.0 kg/m^2^), and obesity (BMI≥28.0 kg/m^2^) [27]. MetS was defined as having 2 or more abnormalities of the following components based on the modified harmonized International Diabetes Federation (IDF) criteria [5], (1) systolic blood pressure (BP) ≥ 130 mmHg, diastolic BP ≥ 85 mmHg, use of antihypertension medication, or self-reported history of hypertension; (2) fasting blood glucose ≥ 5.6 mmol/L (100 mg/dL), current use of anti-diabetic medication, or self-reported history of diabetes; (3) triglycerides ≥ 1.7 mmol/L (150 mg/dL) or current use of lipid-lowering medication; (4) high-density lipoprotein cholesterol < 1.0 mmol/L (40 mg/dL) for men and < 1.3 mmol/L (50 mg/dL) for women. WC was not included in the definition of MetS, due to its collinearity with BMI [28].

Combing the BMI categories and metabolic health status together, participants were then divided into six groups: MH-NW, MH-OW, MHO, MUH-NW, MUH-OW, and MUO.

### Measurement of baPWV

Bilateral baPWV was evaluated by utilizing an automatic arteriosclerosis detection device (BP-203RPE III; Omron Healthcare Co., Kyoto, Japan). Information of the participants was recorded prior to the measurement, including age, sex, height, and weight. Before the examination, participants should stay away from cigarettes, caffeinated or alcoholic beverages for at least 3 h and have a minimum resting time of 5 min in a supine position. Cuffs were attached to both the upper arms and ankles with certain strain. The lower border of the branchial cuff was tied 2-3 cm above the cubital fossa transverse, and the lower border of the ankle cuff was tied 1-2 cm above the medial malleolus. The cardiechema collecting device was placed at the left border of the sternum, with electrodes clipping to both waists for electrocardiography acquisition. The measurement of baPWV was repeated twice by trained nurses, and the second value was recorded. The maximum value of left- and right-side baPWV was used in further analysis. BaPWV ≥1800 cm/s was considered as arterial stiffness [29]. Moreover, the second measurement of baPWV was performed during the two-year interval follow-ups. The change of baPWV was calculated as re-examined baPWV subtracting baseline baPWV, and the new occurrence of baPWV abnormality was defined as normal baPWV at baseline but abnormal baPWV at follow-up.

### Other baseline measurements

Data on demographic characteristics as age, sex, education level, average income, smoking status, drinking status, physical activity, salt intake, past medical history (including hypertension, diabetes, dyslipidemia, myocardial infarction, stroke), and current medication were self-reported on a questionnaire at baseline. WC and BP were measured on admission. Fasting glucose, total cholesterol, triglycerides, low-density lipoprotein, high-density lipoprotein, and C-reactive protein were analyzed by an auto-analyzer (Hitachi 747; Hitachi, Tokyo, Japan) at the central laboratory of the Kailuan hospital.

### Statistical analysis

Continuous variables were expressed as mean ± standard deviation (SD) or median (interquartile range, IQR), categorical variables were presented as count (percentage). The ANOVA or nonparametric Kruskal-Wallis test was used to compare group differences for continuous variables, and χ^2^ test was used for categories variables.

Linear and logistic regression analyses were used to assess the association between BMI-metabolic status phenotypes and baseline baPWV in mono-factor and multi-factor models. To verify the causality of obesity status or metabolic syndrome on baPWV, indicated as the change of baPWV or the new occurrence of arterial stiffness, we further performed linear and logistic regression models in participants of the longitudinal study with β coefficients and ORs calculated. Multiple regression models were run as follows. Model 1 was adjusted for age and sex. Model 2 was adjusted for variates in model 1 plus educational level, average income, smoking, drinking, physical activity, sodium intake, history of myocardial infarction, and history of stroke. Model 3 was further adjusted for C-reactive protein. Inverse probability weighting (IPW) was used to minimize selection bias. Weights were based on results from a model of follow-up status, estimated using logistic regression with being followed up or not as the dependent variables and atherogenic risk factors as independent variables. We used the multivariate logistic regression analyses, before and after IPW, to assess whether BMI-MetS phenotypes were associated with higher odds of the change or new occurrence of baPWV abnormality. Sensitivity analyses excluding participants with a history of CVD in both cross-sectional and longitudinal analyses were conducted. Additionally, stratified analysis was performed to assess the cross-sectional as well as the longitudinal association between BMI and metabolic health status. All statistical analyses were performed using SAS software, version 9.4 (SAS Institute, Cary, NC, USA), and a 2-sided value of *P*<0.05 was considered statistically significant.

## Supplementary Material

Supplementary Table 1
