# Supplementary material for: Risk of arterial stiffness according to metabolically healthy obese phenotype: a combined cross-sectional and longitudinal study in kailuan cohort
Source: Aging (Albany NY). 2021 Jun 3;13(11):15114–25. doi: 10.18632/aging.203075 (PMC8221340; doi:10.18632/aging.203075)
Supplement: Supplementary Table 1 [file aging-13-203075-s001.pdf]

SUPPLEMENTARY TABLE

Supplementary Table 1. Characteristics of follow-up and lost-to-follow-up participants.

	Participants with baseline baPWV	Follow-up participants	Lost-to-follow-up participants	Std[a], %
n (%)	37180	16236 (43.67)	20944 (56.33)	
Age, years	52.84 ± 11.83	54.55 ± 11.40	53.84 ± 12.05	19.58
Male, n (%)	26327 (70.81)	10020 (61.71)	16307 (77.86)	9.14
High school or above, n (%)	13656 (36.73)	6767 (41.68)	6889 (32.89)	16.72
Average income ≥ ¥1000/month, n (%)	20905 (56.23)	9644 (59.40)	11261 (53.77)	18.06
Current smoker, n (%)	12568 (33.80)	4992 (30.75)	7576 (36.17)	15.97
Current alcoholic, n (%)	13981 (37.60)	5828 (35.90)	8153 (38.93)	5.46
Physical activity ≥ 3 times/week, n (%)	5331 (14.34)	2428 (14.95)	2903 (13.86)	15.82
Salt intake ≥12 g/day, n (%)	3970 (10.68)	1768 (10.89)	2202 (10.51)	20.45
BMI, kg/m^2	25.14 ± 3.40	24.97 ± 3.42	25.28 ± 3.28	16.55
WC, cm	87.15 ± 10.25	86.11 ± 10.25	87.95 ± 10.18	1.54
Systolic blood pressure, mmHg	128.23 ± 18.58	126.10 ± 18.40	129.88 ± 18.56	9.44
Diastolic blood pressure, mmHg	83.51 ± 10.93	82.49 ± 11.03	84.29 ± 10.79	5.77
Fasting glucose, mmol/L	5.68 ± 1.57	5.54 ± 1.40	5.79 ± 1.69	6.50
Total cholesterol, mmol/L	5.00 ± 1.29	4.99 ± 1.44	5.01 ± 1.16	23.34
Triglycerides, mmol/L	1.70 ± 1.26	1.66 ± 1.26	1.73 ± 1.27	35.72
LDL-cholesterol, mmol/L	2.60 ± 0.97	2.54 ± 0.79	2.63 ± 1.09	35.72
HDL-cholesterol, mmol/L	1.54 ± 0.45	1.58 ± 0.46	1.51 ± 0.44	9.48
CRP, mg/L	2.16 ± 4.60	1.99 ± 4.76	2.29 ± 4.47	13.74
Hypertension, n (%)	6179 (16.62)	2778 (17.11)	3401 (16.24)	18.78
Hypertension medication, n (%)	4662 (12.54)	2046 (12.60)	2616 (12.49)	4.62
Diabetes, n (%)	2097 (5.64)	882 (5.43)	1215 (5.80)	14.47
Diabetes medication, n (%)	1652 (4.44)	663 (4.08)	989 (4.72)	15.74
Dyslipidemia, n (%)	2445 (6.58)	1131 (6.97)	1314 (6.27)	1.92
Dyslipidemia medication, n (%)	782 (2.10)	377 (2.32)	405 (1.93)	11.52
History of MI	358 (0.96)	127 (0.78)	231 (1.10)	6.27
History of stroke	488 (1.31)	155 (0.95)	333 (1.59)	18.25

Values are (%) for categorical variables and mean ± SD or median (IQR) for continuous variables; baPWV, branchial-ankle pulse wave velocity; Std, standardized difference; BMI, body mass index; WC, waist circumstance; LDL, low-density lipoprotein; HDL, high-density lipoprotein; CRP, C-reactive protein.

Std was calculated as the differences in means or proportions divided by a pooled estimate of the standard deviation, greater than 10% is considered a significant difference between two groups.
